# Effectiveness of multifaceted implementation strategies for the implementation of back and neck pain guidelines in health care: a systematic review

**DOI:** 10.1186/s13012-016-0482-7

**Published:** 2016-09-20

**Authors:** Arnela Suman, Marije F. Dikkers, Frederieke G. Schaafsma, Maurits W. van Tulder, Johannes R. Anema

**Affiliations:** 1 Department of Public and Occupational Health, VU University medical centre and the EMGO+ Institute for Health and Care Research, PO Box 7057, 1007 MB Amsterdam, The Netherlands; 2 Department of Health Sciences, Faculty of Earth and Life Sciences, VU University, Amsterdam, The Netherlands; 3 Research Centre for Insurance Medicine, Collaboration between AMC-UMCG-UWV-VUmc, Amsterdam, The Netherlands

**Keywords:** Systematic review, Multifaceted implementation strategies, Guideline implementation, Non-specific low back pain

## Abstract

**Background:**

For the optimal use of clinical guidelines in daily practice, mere distribution of guidelines and materials is not enough, and active implementation is needed. This review investigated the effectiveness of multifaceted implementation strategies compared to minimal, single, or no implementation strategy for the implementation of non-specific low back and/or neck pain guidelines in health care.

**Methods:**

The following electronic databases were searched from inception to June 1, 2015: MEDLINE, Embase, PsycInfo, the Cochrane Library, and CINAHL. The search strategy was restricted to low back pain, neck pain, and implementation research. Studies were included if their design was a randomized controlled trial, reporting on patients (age ≥18 years) with non-specific low back pain or neck pain (with or without radiating pain). Trials were eligible if they reported patient outcomes, measures of healthcare professional behaviour, and/or outcomes on healthcare level. The primary outcome was professional behaviour. Guidelines that were evaluated in the studies had to be implemented in a healthcare setting. No language restrictions were applied, and studies had to be published full-text in peer-reviewed journals, thus excluding abstract only publications, conference abstracts, and dissertation articles. Two researchers independently screened titles and abstract, extracted data from included studies, and performed risk of bias assessments.

**Results:**

After removal of duplicates, the search resulted in 4750 abstracts to be screened. Of 43 full-text articles assessed for eligibility, 12 were included in this review, reporting on 9 individual studies, and separate cost-effectiveness analyses of 3 included studies. Implementation strategies varied between studies. Meta-analyses did not reveal any differences in effect between multifaceted strategies and controls.

**Conclusion:**

This review showed that multifaceted strategies for the implementation of neck and/or back pain guidelines in health care do not significantly improve professional behaviour outcomes. No effects on patient outcomes or cost of care could be found. More research is necessary to determine whether multifaceted implementation strategies are conducted as planned and whether these strategies are effective in changing professional behaviour and thereby clinical practice.

**Electronic supplementary material:**

The online version of this article (doi:10.1186/s13012-016-0482-7) contains supplementary material, which is available to authorized users.

## Introduction

The recent Global Burden of Disease Study showed that low back pain (LBP), with 83 million years lived with disability, is the leading cause of disability worldwide, while neck pain (NP) is ranked 4th with 33.6 million years lived with disability [1–3]. To assist healthcare professionals in providing best-evidence care for LBP and NP, many guidelines for these health problems have been developed [4, 5]. Clinical practice guidelines are defined by the Institute of Medicine as ‘statements that include recommendations intended to optimize patient care that are informed by a systematic review of evidence and an assessment of the benefits and harms of alternative care options, and are aimed at improving healthcare quality and outcomes’ [6]. Most of the guidelines for LBP/NP are developed for multidisciplinary use in primary care and are mainly national professional guidelines. The contents of these guidelines are similar. For example, the guidelines encourage similar diagnostic triages and discourage the use of diagnostic imaging, bed rest, and referrals to specialist care unless neurological or pathological causes are suspected [4]. The use of these guidelines might improve the quality of care for patients with LBP/NP and reduce the financial and societal burden of these disorders.

For the optimal use of guidelines in clinical practice, mere distribution of the guideline and information materials among healthcare professionals is not enough and active implementation is a necessity [7]. Many studies have been conducted to investigate the effectiveness of implementation strategies. For example, the Effective Practice and Organisation of Care (EPOC) Group of the Cochrane Collaboration has published several systematic reviews on this topic. The results of various implementation strategies, such as the use of educational meetings and workshops, educational outreach, and audit and feedback have shown small effects on improvement of professional practice (6 % improvement for educational meetings and outreach and 5 % for audit and feedback) [8–10]. In line with these findings, Grol and Wensing argued that the simultaneous use of several implementation strategies, i.e. a multifaceted or multicomponent approach to implementation is most effective in successfully implementing guidelines and thus changing practice [7]. However, a recent overview of systematic reviews of multifaceted implementation strategies by Squires et al. suggested that these strategies may not be more effective than single-component interventions [11].

As no studies up to now specifically reviewed the effect of multifaceted implementation strategies for the implementation of non-specific LBP/NP guidelines, the current systematic review will address the following research question: ‘What is the effectiveness of multifaceted implementation strategies compared to minimal, single or no implementation strategy for the implementation of non-specific low back and/or neck pain guidelines in health care?’ Outcomes on healthcare professional behaviour (e.g. referral for diagnostic imaging), and patient health (e.g. quality of life) will be assessed to measure adherence to the guidelines and thereby the success of the implementation process.

## Material and methods

### Eligibility criteria

For the purpose of this review, multifaceted strategies were defined as interventions that consist of a combination of two or more elements from the implementation strategy taxonomy of the EPOC classification system [12]. As this review aimed to assess the effectiveness of implementation strategies, studies were considered for this review if their design was a randomized controlled trial (RCT), involving either individual or cluster randomization and including a control group that received a minimal, single, or no implementation strategy. Studies were eligible if reporting on patients of either gender (age ≥18 years) with non-specific LBP or NP (with or without radiating pain) of any duration. Studies of LBP or NP caused by infection, cauda equina syndrome, bone rarefaction, compression fracture of a vertebral body, tumour, or fibromyalgia were excluded. Cost-effectiveness analyses of included trials were also included. Trials were eligible if they reported measures of healthcare professional behaviour (the primary outcome for this review, patient outcomes, and/or outcomes on healthcare level). Guidelines that were evaluated in the studies had to be implemented in a healthcare setting (i.e. a setting where individual health care is provided to a patient), for example, primary care (general practitioner (GP) or physiotherapist (PT)), occupational health care, or secondary (hospital) care. Guidelines for healthcare insurance were therefore excluded. No language restrictions were applied, and studies had to be published full-text in peer-reviewed journals, thus excluding abstract only publications, conference abstracts, and dissertation articles.

### Information sources

The following electronic databases were searched until June 1, 2015: MEDLINE (PubMed), Embase, PsycInfo, the Cochrane Library, and CINAHL (Ebsco). In close collaboration with a medical information specialist, a broad search was performed with only two restrictions: LBP and/or NP and implementation. Full electronic search strategies for all five databases are presented in Additional file 1: Appendix A. In cases of ambiguity, or where full-text publications of selected abstracts could not be found, authors of the respective studies were contacted. The reference lists of all included studies were screened to identify additional studies.

### Study selection and data extraction

After removal of duplicate results, two reviewers (AS and MD) independently reviewed all titles and abstracts identified by the electronic search. Subsequently, the reviewers engaged in a consensus method to eliminate discrepancies in the selection process. In cases where the reviewers could not come to consensus regarding study eligibility, a third reviewer (FGS or MWvT) was consulted. Full-text articles of studies were obtained when the study was deemed to meet the inclusion criteria or in cases where perusal of title and abstract did not provide sufficient information to assess eligibility of the study. Both reviewers independently screened all selected full-text articles for definitive eligibility, and the same consensus protocol was followed as for the screening of titles and abstracts. Using an adapted form of the ‘Good practice data extraction form’ of the EPOC group, study characteristics and relevant data of all included studies were independently extracted by the two reviewers (AS and MD). Results were discussed in order to reach consensus and assure correct interpretation of the studies. In cases where consensus could not be reached, a third reviewer (FGS) was consulted.

### Assessment of risk of bias of studies

The risk of bias of the included randomized trials was evaluated by two review authors independently (AS and MD), using the Cochrane Collaboration’s tool for assessing risk of bias and the suggested risk of bias criteria for EPOC reviews [13, 14]. Disagreements were resolved by consensus. The quality of the economic evaluations was not assessed, because this was outside the scope of the current review. The following criteria were assessed for high, unclear or low risk of bias for every study: random sequence generation (selection bias); allocation concealment (selection bias); similarity of baseline characteristics and outcome measurements; follow-up; blinding of participants and personnel (performance bias); blinding of outcome assessment (detection bias); protection against contamination; incomplete outcome data (attrition bias); selective outcome reporting (reporting bias); and other bias.

Studies that had a low risk of bias score on at least six criteria were judged to be low risk of bias studies [15]. Studies that had five or less low risk of bias scores were judged to be high risk of bias studies.

Two review authors (AS and MD) independently assessed the overall quality of the evidence for all pooled outcomes using the Grading of Recommendations Assessment, Development and Evaluation (GRADE) approach [16–19]. The GRADE approach specifies four levels of quality. High-quality rating is for randomized controlled evidence, and the quality rating can be downgraded if limitations in one or more of the following domains are encountered: Study limitations encountered in ‘risk of bias’ assessment of study; consistency of study (i.e. the similarity of estimates of treatment effects for the outcome across studies); directness of the study (i.e. the extent to which the participants, interventions, and outcomes in the studies were comparable to those defined in the inclusion criteria of this review); precision of the study (i.e. the degree of certainty surrounding an effect estimate); and publication bias (i.e. the probability of selective publication of studies and outcomes).

The overall quality of the evidence for each pooled outcome was the result of the combination of all domains and leads to four levels of evidence [18]:

- High-quality evidence: Further research is very unlikely to change the confidence in the estimate of effect.
- Moderate-quality evidence: Further research is likely to have an impact on the confidence in the estimate of effect and may change the estimate.
- Low-quality evidence: Further research is very likely to have an important impact on the confidence in the estimate of effect and is likely to change the estimate.
- Very low-quality evidence: Any estimate of effect is very uncertain.

### Data extraction

Two independent reviewers (AS and MD) extracted data from the included studies using the EPOC data collection checklist and data extraction template [12]. The data extraction form was the first pilot tested using one of the included studies. Disagreements in data extraction were resolved by consensus. The following data were extracted:

- Bibliographic data (authors, title study, journal, and date of publication)
- Study characteristics (study type and design, unit of allocation, duration of follow-up)
- Participant characteristics (population description (e.g. (neck or back pain) patients or (type of) professionals), total number of participants randomized, mean age, gender, severity of illness, co-morbidities)
- Setting characteristics (location, social context, clusters, withdrawals, and exclusions)
- Description of intervention and control groups (content, dose, components, duration, timing, delivery, providers, number randomized to group, theory base)
- Outcomes assessed (outcome definitions, time points measured and reported, unit of measurement, outcome tool, scales, missing data)
- Study results (baseline data, comparison, outcome, subgroup, time points, results intervention and comparison)

### Synthesis of results

The included studies first were categorized into types of interventions (according to the EPOC taxonomy) and types of outcome measures. Meta-analyses were separately planned and conducted for the comparison of multifaceted implementation strategies vs. controls (i.e. usual care or minimal implementation) for various outcomes. Outcomes on healthcare professional behaviour were considered indicators for guideline adherence and thus the primary outcome for success of guideline implementation. The effects on professional behaviour were categorized into ‘treatment’ and ‘referral’ behaviour outcome groups. In the treatment group, outcomes on treatment behaviour were classified into adequate patient information, advising active treatment, and prescribing medication. Outcomes on referral behaviour were separately analysed for referrals for X-ray, computed tomography (CT), or magnetic resonance imaging (MRI) scans, physical therapy, and speciality/secondary care referrals. Additional file 2: Appendix C shows the data sources and calculations used for the meta-analyses.

To calculate effects, the data for the pooled outcomes of each study were entered into Review Manager (RevMan) 5.3 software. All pooled outcome data were dichotomous or dichotomized, and for all outcomes, odds ratios (using random effects models) and 95 % CIs were calculated in RevMan to estimate the implementation effects. To determine the presence of heterogeneity, *I* ^2^ was analysed in RevMan. When *I* ^2^ was more than 50 %, the studies were judged to be heterogeneous.

## Results

### Identification and selection of studies

**Fig. 1:**
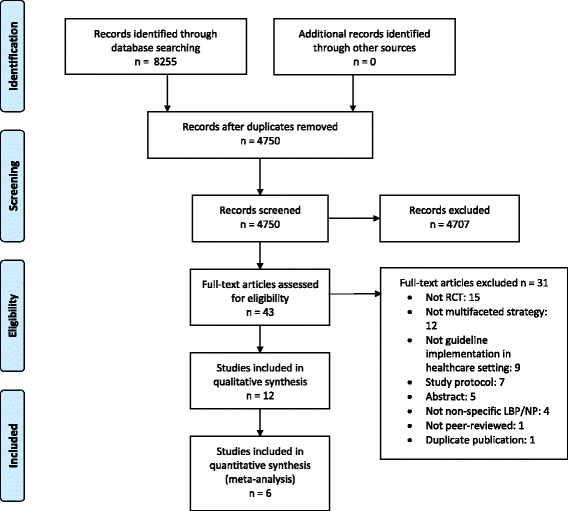
Flowchart of inclusion process

The electronic search resulted in 8255 references, of which 2476 were retrieved from MEDLINE, 4181 from Embase, 876 from CINAHL, 293 from Cochrane, and 429 from PsycInfo. After removing duplicates, titles and abstracts of 4750 records were screened. Of 43 records, full-text articles were screened for eligibility. Twelve articles were included in the current review (see Additional file 3: Table S1) [20–31], and 31 articles were excluded (see Additional file 4: Table S2) [32–62]. Figure 1 shows a flow chart of the inclusion process, including reasons for exclusion (several exclusion reasons per article possible) of records. Screening of reference lists of the included articles did not result in any additional inclusions.

The 12 included articles were based on 8 individual studies, of which 4 based their implementation strategy on theory and described the development of their strategy. Three studies separately reported cost-effectiveness analyses, and 1 study described outcomes on patient and professional levels in 2 separate articles. One study was targeted at patients with a whiplash, while all other studies were on non-specific LBP. Additional file 5: Table S3 provides a summary of the included studies and their characteristics. A great variety of intervention elements was applied across the studies, and several outcomes were measured. The guidelines implemented in the included studies had similar objectives such as encouraging activation, restoration of normal functioning, and exercise, while discouraging referrals for secondary care and diagnostic imaging. Additional file 6: Appendix B lists the excluded full-text articles with reasons for their exclusion.

In Additional file 7: Table S4, intervention elements according to the EPOC taxonomy are shown for the included studies. Nine types of elements could be identified. Next to the obvious dissemination of clinical practice guidelines, educational material and educational meetings were the most commonly applied elements. Local opinion leaders, audit and feedback, reminders, and organizational interventions were not used as often, and only three studies applied a patient-mediated intervention element. The implementation strategies of most studies consisted of four to five intervention elements. Additional file 8: Table S5 shows the types of outcomes that were measured in the included studies. Most studies measured physician treatment adherence to guideline recommendations and the number of referrals to secondary care, medical diagnostics, and/or physical therapy. Only three studies reported outcomes on patient level.

### Quality of included studies

**Fig. 2:**
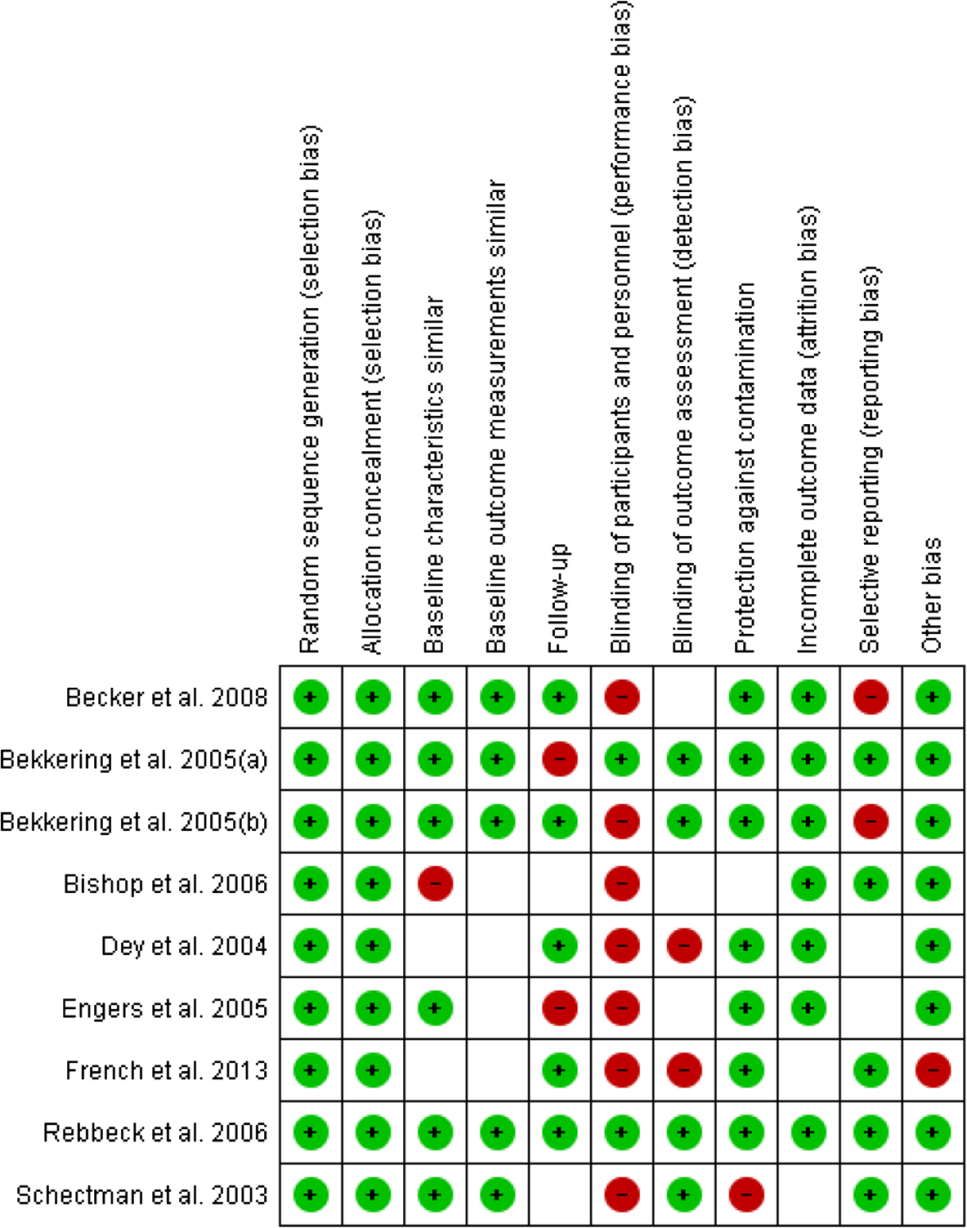
Risk of bias for included studies

Figure 2 shows the risk of bias judgement of the included randomized trials. With only two studies [25, 28] judged to have a high risk of bias according to the predefined cut-off point, overall quality of the included studies was good. Blinding of participants and personnel (performance bias) was judged to be a source of high risk of bias in all but two studies [22, 30]. Other sources of bias like a follow-up of at least 80 %, blinding of outcome assessment, and selective reporting were considered a risk of bias in a few studies only. For four of the nine studies, the risk of bias based on similarity of baseline outcome measurements was unclear. Additional file 9: Table S6 shows the summary of findings table, including the assessment of the quality of the evidence using the GRADE system.

### Effect on professional behaviour

#### Referral behaviour

**Fig. 3:**
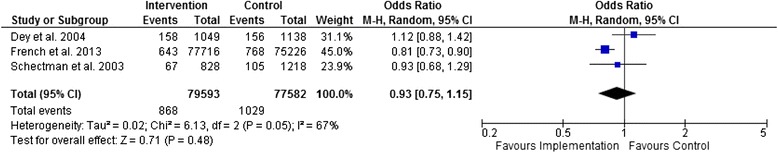
Pooled analysis for referral rates for X-rays (moderate-quality evidence)

**Fig. 4:**
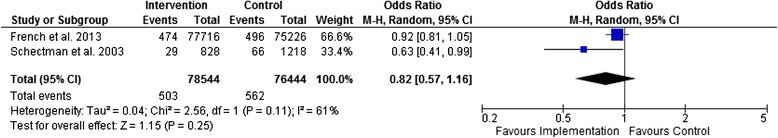
Pooled analysis for referral rates for CT/MRI scans (low-quality evidence)

**Fig. 5:**
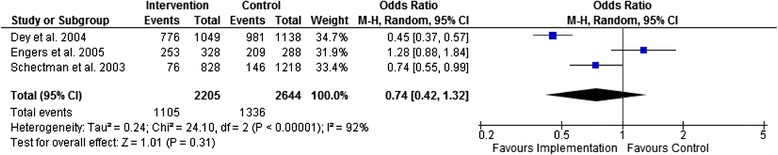
Pooled analysis for referral rates for physiotherapy (very low-quality evidence)

**Fig. 6:**
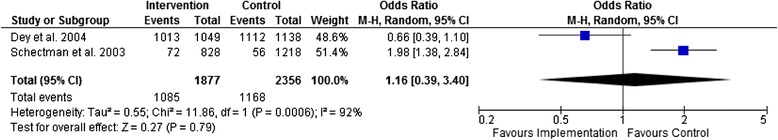
Pooled analysis for referral rates for secondary/specialty care (very low-quality evidence)

Figures 3, 4, 5, and 6 show that multifaceted implementation is not more effective than usual care or minimal implementation in improving guideline concordant referral behaviour. The statistical heterogeneity of the pooled studies is high.

#### Treatment behaviour

**Fig. 7:**
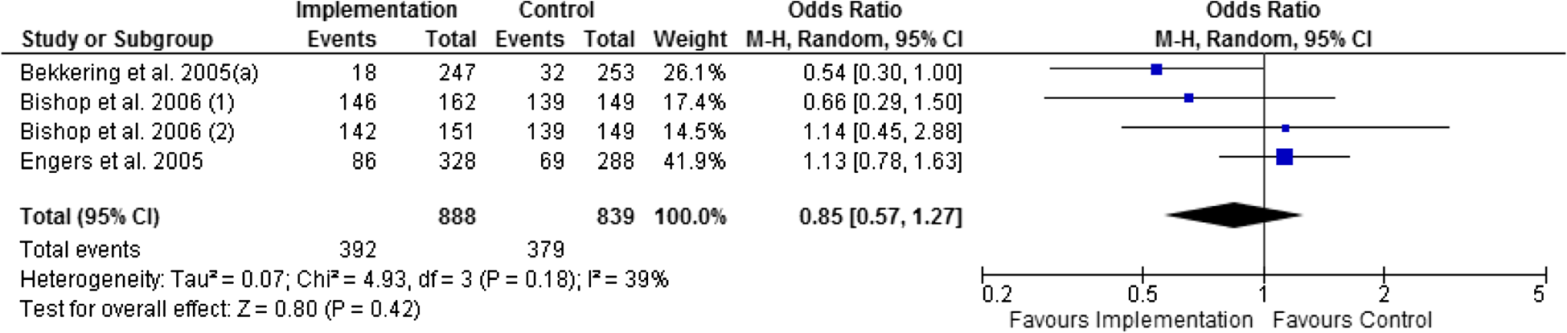
Pooled analysis for the provision of adequate patient information (low-quality evidence)

**Fig. 8:**
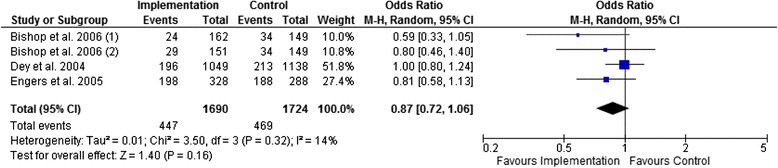
Pooled analysis for medication prescription (low-quality evidence)

**Fig. 9:**
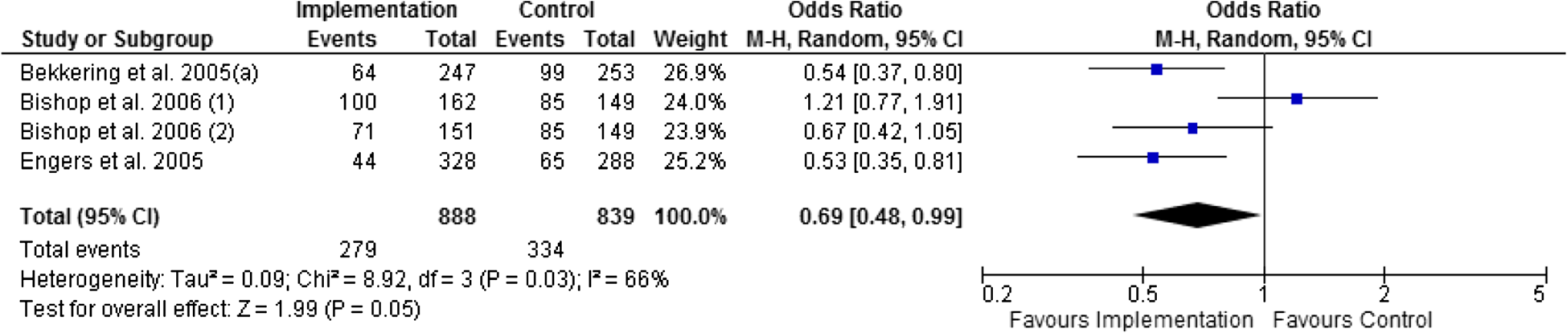
Pooled analysis for advising active treatment (low-quality evidence)

Figures 7, 8, and 9 show that there is no statistically significant difference between multifaceted implementation and usual care or minimal implementation in providing adequate patient information and prescribing medication. However, active treatment was more often advised in the multifaceted implementation groups than in the control groups (Fig. 9, OR 0.69; 95 % CI 0.48 to 0.99).

### Effect on patient outcomes

Additional file 10: Table S7 shows the results for patient outcomes at 12-month follow-up. Three studies reported outcomes on patient level [20, 23, 30]. The most common patient outcomes measured were functional capacity or disability, days of sick leave, and quality of life (QoL). No significant differences on 12-month follow-up were found for any of these outcomes.

### Effect on cost of care

Three studies performed and separately reported a cost-effectiveness analysis for their implementation strategies [21, 24, 29]. Two of these cost-effectiveness analyses [21, 29] showed that multifaceted implementation yielded lower costs and more effects, although these results were not statistically significant. The third study showed no cost or effect advantages for the intervention group compared to the control group.

## Discussion

This review showed that multifaceted strategies for the implementation of neck and/or back pain guidelines in health care do not significantly improve professional behaviour outcomes. Only active treatment was more often advised in the multifaceted implementation groups than in the control groups. No effects on patient outcomes or cost of care could be found. These results are not in line with findings from previous research in other fields that showed that active, multifaceted implementation strategies are effective in changing professional behaviour [63–69] compared to passive dissemination of guidelines or minimal implementation activities. However, the results are in line with a more recent and more elaborate overview of systematic reviews, which suggested that multifaceted implementation strategies are not more effective than other strategies [11].

Few studies that were found in the electronic database search were included in the current review. Many studies were excluded because their interpretations of ‘multifaceted’ strategies were not in line with the EPOC definition. For example, several studies indicated having applied a multifaceted approach by organizing several workshops on multiple occasions. However, all workshops were part of one element, i.e. educational meetings. As according to the EPOC taxonomy, an intervention is multifaceted if it applies two or more elements [12], these strategies did not meet the criteria for being multifaceted. It seems that either the EPOC taxonomy is not often used or the definition of multifaceted strategies is open to interpretation. Either way, consensus on the definition of multifaceted and application of the taxonomy could improve insight into the effectiveness of multifaceted implementation strategies.

Only 12 articles were identified and included in this study. These studies were not able to produce high-quality evidence for changes on professional or patient outcomes in the current review. Only 3 articles reported cost-effectiveness analyses of original studies. These cost-effectiveness studies were evaluated by a recent review by Jensen et al., who showed that the quality of these economic evaluations was moderate and that the studies, although similar to one another, showed conflicting results on cost-effectiveness [70]. It is advisable that researchers implementing guidelines also include cost-effectiveness in their analyses. Multifaceted implementation strategies can be costly, especially when they are applied to implement guidelines on a national level. The efforts and resources for applying these strategies are only worthwhile if they are effective in improving patient outcomes or quality of care. If these strategies also lead to changes in specific professional behaviour, e.g. less unnecessary healthcare utilization referrals or medication prescriptions, and advises to return-to-work, the costs of implementation might be offset by the decrease in costs of care.

Of the included studies, only 3 applied a patient-mediated implementation element. Of these 3 studies, only 1 actually measured patient outcomes (20). Two other studies reported patient outcomes, while they had not applied patient-mediated elements. This might be an explanation for the lack of effect on patient outcomes. Grol and Wensing [7] identified several patient characteristics as possible factors for implementation success. For example, patient attitude and knowledge might pose a barrier for the uptake of changes by professionals. Therefore, when aiming to improve professional practice, applying elements that are targeted at patient-mediated barriers and facilitators might be essential to guideline implementation. This is underlined by the slightly more positive results in the study of Becker et al. [20] compared to the other studies that reported patient outcomes. However, merely applying patient-mediated interventions does not necessarily address patient barriers. It is advisable that these barriers be taken into account when designing implementation strategies.

Regarding the effectiveness of more comprehensive strategies compared to strategies that apply fewer elements, the results from the current review are inconclusive. It seems that more does not always mean better, and multifaceted strategies possibly are only more effective when they apply different elements that are targeted at various barriers and facilitators for change [71]. However, this was not confirmed in our review. A recent review by Mesner et al. suggested that the success of implementation interventions does not necessarily depend on the specific type of interventions but rather might be determined by the increase of frequency and duration of implementation interventions [72]. There is still a lack of the use of theory in implementation research, and studies on guideline implementation strategies poorly justify the choice of intervention [72, 73]. This is in line with the findings of the current review, in which only four studies (partly) based their strategies on theories and also reported on the development of their strategy. This could be one possible explanation for the lack of effective results in these studies [73]. However, for many studies, it is unknown whether the implementation strategies were conducted as planned, which might be another factor influencing the effectiveness of the implementation strategies. Process evaluations are necessary to gain more insight into this factor; however, of the included studies in this review, only one study performed a process evaluation [28] and reported moderate to high levels of fidelity [74].

### Strengths and limitations of this study

When interpreting the results of the current review, some limitations should be taken into account. Firstly, this review did not search for unpublished studies. Also, due to the amount of synonyms for the term ‘Implementation’ included in the search strategy, it might be possible that some studies were missed during the search phase. Furthermore, all but two studies were published before 2007, and the quality of the evidence found in these studies was very low to moderate, according to the GRADE assessment performed in the current review. Another important limitation is the comparability of the studies. Not only did the studies apply various implementation strategies, usual dissemination in the control groups also varied. Besides, there was a wide variation in the outcomes that were measured, how they were measured, and how they were reported. In the meta-analyses, the statistical heterogeneity was large, and results should therefore be interpreted with caution. For example, one study where the control group did not receive any implementation strategy was compared with a study in which the control group received a patient-mediated implementation strategy. Other reasons for heterogeneity might be clinical (e.g. due to different settings and patients) or statistical (e.g. different study sizes). No sensitivity analyses were performed due to the small number of studies that could be included in the meta-analyses, and notwithstanding the high statistical heterogeneity found in these analyses as expressed by the *I* ^2^ measures, these analyses might give an insight into the effect directions of the included studies.

By following the method guidelines for systematic reviews as posed by the Cochrane Back Review Group [75], and the Cochrane Handbook for Systematic Reviews of Interventions [12], the current review pursued the highest methodological quality. By applying a broad and comprehensive search strategy and supplementary hand search of reference lists of included studies, this review ensured that as few as possible, eligible studies were missed. To further minimize this chance, no language restrictions were applied during the inclusion and data collection phases.

## Conclusion

This review showed that multifaceted strategies for the implementation of neck and/or back pain guidelines in health care do not significantly improve professional behaviour outcomes. Only active treatment was more often advised in the multifaceted implementation groups than in the control groups. No effects on patient outcomes or cost of care could be found.

## Additional files

Additional file 1: Appendix A. Full electronic database searches. (DOCX 14 kb)

Additional file 2: Appendix C. Data sources and calculations for meta-analyses. (DOCX 15 kb)

Additional file 3: Table S1. References to included studies. (DOCX 15 kb)

Additional file 4: Table S2. References to excluded full-text studies. (DOCX 17 kb)

Additional file 5: Table S3. Characteristics of included studies. (DOCX 23 kb)

Additional file 6: Appendix B. List of excluded studies and reasons for exclusion. (DOCX 17 kb)

Additional file 7: Table S4. Interventions of included studies according to EPOC taxonomy (all on professional level unless stated otherwise). (DOCX 15 kb)

Additional file 8: Table S5. Grouped outcome measures of included studies (all on professional level unless stated otherwise). (DOCX 14 kb)

Additional file 9: Table S6. Summary of findings. (DOCX 17 kb)

Additional file 10: Table S7. Patient outcomes at 12-month follow-up. (DOCX 14 kb)

## Abbreviations

CEA: Cost-effectiveness analysis
CT: Computed tomography
EPOC: Effective Practice and Organisation of Care
GP: General practitioner
LBP: Low back pain
MRI: Magnetic resonance imaging
NP: Neck pain
NPs: Nurse practitioners
PAs: Physician assistants
PT: Physiotherapist
QoL: Quality of life
RCT: Randomized controlled trial
X-ray: Röntgen radiation imaging
