# Supplementary material for: Effectiveness of multifaceted implementation strategies for the implementation of back and neck pain guidelines in health care: a systematic review
Source: Implement Sci. 2016 Sep 20;11:126. doi: 10.1186/s13012-016-0482-7 (PMC5029102; doi:10.1186/s13012-016-0482-7)
Supplement: Supplementary file 1 — Appendix A. Full electronic database searches. (DOCX 14 kb) [file 13012_2016_482_MOESM1_ESM.docx]

**Additional file 1: Table S1. References to included studies**

| **ID** | **Study** |
| --- | --- |
| I1 | Becker A, Leonhardt C, Kochen MM, Keller S, Wegscheider K, Baum E, et al. Effects of Two Guideline Implementation Strategies on Patient Outcomes in Primary Care. A Cluster Randomized Controlled Trial. SPINE. 2008; 33(5):4732-480. |
| I2 | Becker A, Held H, Redaelli M, Chenot JF, Leonhardt C, Keller S, et al. Implementation of a Guideline for Low Back Pain Management in Primary Care. A Cost-Effectiveness Analysis. SPINE. 2012; 37(8):701-710. |
| I3 | Bekkering GE, Hendriks HJM, van Tulder MW, Knol DL, Hoeijenbos M, Oostendorp RAB. Effect on the process of care of an active strategy to implement clinical guidelines on physiotherapy for low back pain: a cluster randomised controlled trial. Qual Saf Health Care. 2005; 14:107-112. |
| I4 | Bekkering GE, van Tulder MW, Hendriks HJM, Koopmanschap MA, Knol DL, Bouter LM, et al. Implementation of Clinical Guidelines on Physical Therapy for Patients With Low Back Pain: Randomized Trial Comparing Patient Outcomes After a Standard and Active Implementation Strategy. Phys Ther. 2005; 85:544-555. |
| I5 | Hoeijenbos M, Bekkering T, Lamers L, Hendriks E, van Tulder M, Koopmanschap M. Cost-effectiveness of an active implementation strategy for the Dutch physiotherapy guideline for low back pain. Health Policy. 2005; 75:85-98. |
| I6 | Bishop PB, Wing PC. Knowledge transfer in family physicians managing patients with acute low back pain: a prospective randomized control trial. The Spine Journal. 2006; 6:282-288. |
| I7 | Dey P, Simpson CWR, Collins SI, Hodgson G, Dowrick CF, Simison AJM, et al. Implementation of RCGP guidelines for acute low back pain: a cluster randomised controlled trial. British Journal of General Practice. 2004; 54:33-37. |
| I8 | Engers AJ, Wensing M, van Tulder MW, Timmermans A, Oostendorp RAB, Koes BW, et al. Implementation of the Dutch Low Back Pain Guideline for General Practitioners. SPINE. 2005; 6:595-600. |
| I9 | French SD, McKenzie JE, O’Connor DA, Grimshaw JM, Mortimer D, Francis JJ, et al. Evaluation of a Theory-Informed Implementation Intervention for the Management of Acute Low Back Pain in General Medical Practice: The IMPLEMENT Cluster Randomised Trial. PLoS ONE. 2013; 8(6):e65471; doi:10.1371/journal.pone.0065471. |
| I10 | Mortimer D, French SD, McKenzie JE, O’Connor DA, Green SE. Economic Evaluation of Active Implementation versus Guideline Dissemination for Evidence-Based Care of Acute Low-Back Pain in a General Practice Setting. PLoS ONE. 2013; 8(10):e75647; doi:10.1371/journal.pone.0075647. |
| I11 | Rebbeck T, Maher CG, Refshauge KM. Evaluation two implementation strategies for whiplash guidelines in physiotherapy: A cluster-randomised trial. Australian Journal of Physiotherapy. 2006; 52:165-174. |
| I12 | Schectman JM, Schroth WS, Verme D, Voss JD. Randomized Controlled Trial of Education and Feedback for Implementation of Guidelines for Acute Low Back Pain. J Gen Intern Med. 2003; 18:773-780. |
