# Supplementary material for: Effectiveness of multifaceted implementation strategies for the implementation of back and neck pain guidelines in health care: a systematic review
Source: Implement Sci. 2016 Sep 20;11:126. doi: 10.1186/s13012-016-0482-7 (PMC5029102; doi:10.1186/s13012-016-0482-7)
Supplement: Supplementary file 2 — Appendix C. Data sources and calculations for meta-analyses. (DOCX 15 kb) [file 13012_2016_482_MOESM2_ESM.docx]

**Additional file 2: Table S2. References to excluded full-text studies**

| **ID** | **Study** |
| --- | --- |
| E1 | Ammendolia C, Hogg-Johnson S, Pennick V, Glazier R, Bombardier C. Implementing evidence-based guidelines for radiography in acute low back pain: a pilot study in a chiropractic community. Journal of Manipulative and Physiological Therapeutics. 2003; 27(3):170-179. |
| E2 | Bussières A, Laurencelle L, Peterson C. Diagnostic Imaging Guidelines Implementation Study for Spinal Disorders. A Randomized Trial with Postal Follow-ups. The Journal of Chiropractic Education. 2010; 24(1). |
| E3 | Bekkering GE, Hendriks HJM, Van Tulder MW. Effectiviteit van een actieve implementatiestrategie van de KNGF-richtlijn ‘Lage-rugpijn’. Ned Tijdschr Fysiother. 2005; 115(3):62-67. |
| E4 | Cherkin D, Deyo RA, Berg AO, Bergman JJ, Lishner DM. Evaluation of a Physician Education Intervention to Improve Primary Care for Low-Back Pain 1: Impact on Physicians. SPINE. 1991; 16(10): 1168-1172. |
| E5 | Cunningham CG, Flynn TA, Toole CM, Ryan RG, Gueret PWJ, Bulfin S, et al. Working Backs Project – implementing low back pain guidelines. Occupational Medicine. 2008; 58:580-583. |
| E6 | Taramona Espinoza CP, Skupin M, Montezuma D, Sandouk Z, Drake S. Adherence to guidelines for low back pain imaging: comparison of a teaching and non-teaching clinic. Abstracts from the 35^th^ Annual Meeting of the Society of General Internal Medicine. JGIM. 2012; 27”:Suppl 2:S99-574; doi: 10.1007/s11606-012-2038-0. |
| E7 | Evans DW, Foster NE, Underwood M, Vogel S, Breen AC, Pincus T. Testing the effectiveness of an innovative information package on practitioner reported behaviour and beliefs: The UK Chiropractors, Osteopaths and Musculoskeletal Physiotherapists Low back pain ManagemENT (COMPLeMENT) trial. BMC Musculoskeletal Disorders. 2005; 6(41); doi:10.1186/1471-2474-6-41. |
| E8 | Fleuren M, Dusseldorp E, Van den Bergh S, Vlek H, Wildschut J, Van den Akker E, et al. Implementation of a shared care guideline for low back pain: effect on unnecessary referrals. International Journal for Quality in Health Care. 2010; 22(5):415-420. |
| E9 | Jensen CE, Riis A, Pedersen KM, Jensen MB, Petersen KD. Study protocol of an economic evaluation of an extended implementation strategy for the treatment of low back pain in general practice: a cluster randomised controlled trial. Implementation Science. 2014; 9:140. |
| E10 | Lang E, Kastner S, Liebig K, Neundörfer B. Interventions for improvement of primary care in patients with low back pain: how effective are advice to primary care physicians on therapies and a multimodal therapy program arising out of cooperation of outpatient health care structures? Schmerz. 2002; 16:22-33. |
| E11 | McKenzie JE, French SD, O’Connor DA, Grimshaw JM, Mortimer D, Michie S, et al. IMPLEmenting a clinical practice guideline for acute low back pain evidence-based management in general practice (IMPLEMENT): Cluster randomised controlled trial study protocol. Implementation Science. 2008; 3:11; doi:10.1186/1748-5908-3-11. |
| E12 | McKenzie JE, O’Connor DA, Page MJ, Mortimer SD, French SD, Walker BF. Improving the care for people with acute low-back pain by allied health professionals (the ALIGN trial): A cluster randomised trial protocol. Implementation Science. 2013; 5(86). |
| E13 | Mortimer D, French SD, McKenzie JE, O’Connor DA, Green SE. Protocol for economic evaluation alongside the IMPLEMENT cluster randomised controlled trial. Implementation Science. 2008; 3:12; doi:10.1186/1748-5908-3-12. |
| E14 | Rasmussen FØ. Kunnskapsbasert ryggomsorg – en pilotstudie om etterutdanning av allmennleger. Tidsskrift for Den norske legeforening. 2002; 122:1794-6. |
| E15 | Rebbeck TJ, Refshauge KM, Maher CG. Use of clinical guidelines for whiplash by insurers. Aust Health Rev. 2006; 30(4):442-449. |
| E16 | Rebbeck T, Stewart M, Cameron I, Stewart J. Treatment of chronic whiplash: a systematic review and clinical guidelines. Physiotherapy. 2011; 97:s1. |
| E17 | Rebbeck T, Macedo LG, Maher CG. Compliance with clinical guidelines for whiplash improved with a targeted implementation strategy: a prospective cohort study. BMC Health Services Research. 2013; 13(213). |
| E18 | Rebbeck T, Macedo L, Paul P, Trevena L, Camron ID. General practitioners’ knowledge of whiplash guidelines improved with online education. Australian Health Review. 2013; 37:688-694. |
| E19 | Richings K, Taylor C, Morris J. Changing GPS guideline adherence in relation to ordering plain lumbar spine X-rays for NSLBP: the role of the physiotherapist. Physiotherapy. 2011; 97:s1. |
| E20 | Riis A, Jensen CE, Bro F, Maindal HT, Petersen KD, Jensen MB. Enhanced implementation of low back pain guidelines in general practice: study protocol of a cluster randomised controlled trial. Implementation Science. 2013; 8(124). |
| E21 | Rossignol M, Abenhaim L, Séguin P, Neveu A, Collet JP, Ducruet T, et al. Coordination of Primary Health Care for Back Pain: A Randomized Controlled Trial. SPINE. 2000; 25(2):251-259. |
| E22 | Rutten G, Harting J, Bartholomew LK, Oostendorp RAB, De Vries NK. Results of the pilot study of a multilevel intervention to improve adherence to evidence based guidelines for low back pain. Physiotherapy. 2011; 97:s1. |
| E23 | Rutten GM, Harting J, Bartholomew LK, Braspenning J, Van Dolder R, Heijmans MFGJ, et al. Development of a theory- and evidence-based intervention to enhance implementation of physical therapy guidelines for the management of low back pain. Archives of Public Health. 2014; 72(1). |
| E24 | Sandner-Kiesling A, Gspurning E, Granitz GG, Thalhammer G. “Rückenschmerz.ade” – A disease management project for the implementation of guidelines in the treatment of chronic low back pain. European Journal of Pain (Poster Sessions). 2009; 13:S55-S285. |
| E25 | Shenoy S. Cluster randomized controlled trial to evaluate the effectiveness of a multifaceted active strategy to implement low back pain practice guidelines: effect on competence, process of care and patient outcomes in physical therapy. Dissertation at University of Pittsburgh, School of Health and Rehabilitation Sciences, 2013. |
| E26 | Slater H, Briggs AM, Smith AJ, Bunzli S, Davies SJ, Quintner JL. Implementing evidence-informed policy into practice for health care professionals managing people with low back pain in Australian rural settings: a preliminary prospective single-cohort study. Pain Medicine. 2014; 15:1657-1668. |
| E27 | Stiell IG, Clement CM, Grimshaw J, Brison RJ, Rowe BH, Schull MJ, et al. Implementation of the Canadian C-Spine Rule: a prospective 12 centre cluster randomised trial. BMJ. 2009; 339:b4146. |
| E28 | Suman A, Schaafsma FG, Elders PJM, Van Tulder MW, Anema JR. Cost-effectiveness of a multifaceted implementation strategy for the Dutch multidisciplinary guideline for nonspecific low back pain: design of a stepped-wedge cluster randomised controlled trial. BMC Public Health. 2015; 15:522; doi:10.1186/s12889-015-1876-1. |
| E29 | Tracey NG, Martin JB, McKinstry CS, Mathew BM. Guidelines for lumbar spine radiography in acute low back pain: effect of implementation in an accident and emergency department. The Ulster Medical Journal. 1994; 63(1):12-17. |
| E30 | Twomey P. Making the best use of a radiology department: an example of implementation of a referral guideline within a primary care organisation. Quality in Primary Care. 2003; 11:53-9. |
| E31 | Van Dulmen SA, Maas MJ, Staal B, Rutten G, Kiers H, Nijhuis-Van der Sanden M, et al. Effectiveness of Peer Assessment for Implementing a Dutch Physical Therapy Low Back Pain Guideline: cluster randomized controlled trial. PHYS THER. 2014; 94:1396-1409. |
