# Supplementary material for: Effectiveness of multifaceted implementation strategies for the implementation of back and neck pain guidelines in health care: a systematic review
Source: Implement Sci. 2016 Sep 20;11:126. doi: 10.1186/s13012-016-0482-7 (PMC5029102; doi:10.1186/s13012-016-0482-7)
Supplement: Supplementary file 3 — References to included studies. (DOCX 15 kb) [file 13012_2016_482_MOESM3_ESM.docx]

**Additional file 3: Table S3. Characteristics of included studies**

| **Study ID** | **Design & Participants** | **Guideline recommendations** | **Intervention**  **- Theory based?**  **- Development described?** | **Outcomes** | **Authors’ conclusion** |
| --- | --- | --- | --- | --- | --- |
| I1. Becker et al. 2008 | Cluster randomised controlled trial (RCT) with 3 arms  1378 LBP patients  126 General practitioners (GPs)  (118 GP practices) | GP guideline: - Early activation - Symptomatic pain relief - Optional manual therapy - Multidisciplinary treatment (physiotherapy, psychotherapy, back schools, massage) | Implementation group 1: - 3 interactive seminars  - Information packages  - 2 educational visits  Implementation group 2:  - All elements from implementation group 1 + - Motivational counselling strategies  Control: Guideline by mail  Intervention not theory based;  Development of intervention not described | *Patient level:*  1. Functional capacity  2. Days in pain  3. Overall activity  4. Days of sick leave  5. Quality of Life (QoL) | Active implementation of the guideline results in slightly better outcomes during 6 months follow-up that its postal dissemination. Results are more distinct when motivational counselling strategies are additionally applied. |
| I2. Becker et al. 2012 | Cost-effectiveness analysis (CEA) of Becker et al. 2008 (study I1) | See Becker et al. 2008 (I1) | - | *Cost of care:* 1. Absence from work  2. Direct, indirect & total healthcare costs  2. Functional capacity CER  3. Overall activity CER  4. QoL CER | Both implementation groups showed lower direct and indirect costs as well as better patient outcomes during follow-up compared with controls, and showed superiority in cost-effectiveness to control. |
| I3. Bekkering et al. 2005(a) | Cluster RCT  500 LBP patients  113 physiotherapists (PTs) (68 PT practices) | PT guideline: - Active treatment approach - Adequate patient information - Activation - Optional behavioural approach - Restore physical functioning - Improve participation/return to work - Limiting treatment sessions | Implementation: - Guideline by mail - 2 training sessions  Control: Guideline by mail  Intervention theory based; Development of intervention described in separate paper | *Professional level:* 1. Limiting n treatment sessions in patients with normal course  2. Setting functional treatment goals  3.Use active interventions  4. Provide adequate information & advice  5. Adherence to all previous recommendations | The active strategy moderately improved adherence to guidelines. Active strategies are recommended to implement the clinical guidelines on physiotherapy for LBP. |
| I4. Bekkering et al. 2005 (b) | Cluster RCT  500 LBP patients  113 PTs  (68 PT practices) | See Bekkering et al 2005(a) (I3) | Implementation: - Guideline by mail - 2 training sessions  Control: - Guideline by mail  Intervention theory based;  Development of intervention described in separate paper | *Patient level:* 1. Physical Functioning  2. Pain  3. Sick leave (n days off work in last 6 weeks)  4. Pain coping  5. Pain beliefs | Physical functioning and pain improved substantially in the first 12 weeks. No additional benefit to applying active strategy to implement physical therapy guidelines for patients with LBP. Active implementation not recommended if patient outcomes are to be improved. |
| I5. Hoeijenbos et al. 2005 | CEA of Bekkering et al. 2005(a) & (b) (study I3 & I4) | See Bekkering et al 2005(a) (I3) | - | *Cost of care:* 1. Direct medical costs 2. Productivity costs (absenteeism)  3. Efficiency loss 4. Hindrance during unpaid work 5. QoL 6. Annual costs (direct & productivity costs) 7. Cost-effectiveness | During 1-year follow-up, no differences were found in QoL, direct medical costs and productivity costs. Active implementation appears not to be cost effective as compared to standard strategy. |
| I6. Bishop et al. 2006 | Prospective RCT with 3 arms  428 LBP patients | Unknown | Implementation group 1: - Guideline + letter per mail  - Guideline reminders relevant to specific periods of clinical course  Implementation group 2:  - All elements from implementation group 1 +  - Lay language version of guideline for patients (sent to patient)  - Lay language guideline reminder relevant to specific periods of clinical course for patients (sent to patient)  Control: No intervention  Intervention not theory based;  Development of intervention not described | *Professional level:* 1. Adherence to patient assessment 2. Adherence to guideline treatment recommendations | Largely unsuccessful in improving concordance with guideline treatment recommendations. |
| I7. Dey et al. 2004 | Cluster RCT  2187 LBP patients  54 GP practices | GP guideline: - Diagnostic triage for minority of patients - Treatment in primary care - Return to normal function - No use of x-ray, bed rest, secondary care - Recommend simple analgesics and exercise - Optional PT | Implementation: - Educational outreach visit - Poster reinforcing guideline recommendations - Patient text recommended by guideline  - Referral form for fast-track PT  - Referral form for direct access to back clinic  Control: - Poster reinforcing guideline recommendations - Patient text recommended by guideline  Intervention partly based on theory;  Development of intervention described | *Professional level:* 1. Referral for X-ray  2. Issuing of sickness certificate  3. Prescription opioids/muscle relaxants  4. Referral to secondary care  5. Referral for PT or educational programme | No significant differences between groups in proportion of patients referred to X-ray, issues with sickness certificate, prescribed opioids/muscle relaxants, or referred to secondary care. Significantly more patients in implementation group referred to physiotherapy or back clinic. Mostly unchanged management of patients with LBP. |
| I8. Engers et al. 2005 | Cluster RCT  616 consultations for 531 LBP patients  67 GPs | GP guideline: - Activation and treatment - Adequate patient information and advice - Time contingent treatment - Restore normal functioning - Assess psychosocial risk factors - No imaging or lab - Optional non-steroid analgesics | Implementation: - 1 workshop - Patient education card - Guideline for occupational physicians - 2 scientific articles on LBP management by GPs - Collaboration tool for collaboration with PT  Control: No intervention  Intervention theory based;  Development of intervention described | *Professional level:* 1. Number of referrals to a therapist  2. Prescription of time-contingent pain medication  3. Prescription of paracetamol vs. NSAIDS  4. Adequacy of patient education | Implementation strategy modestly improved implementation of the guideline and produced small concomitant changes in patient management. Implementation strategy produced fewer referrals to therapists during follow-up consultations. |
| I9. French et al. 2013 | Cluster RCT  112 GPs  (92 GP practices) | GP guideline: - No x-ray or other imaging - Activation | Implementation: - 2 educational workshops - Film footage of workshop (DVD) - Electronic resources for acute LBP  Control: - Printed copy of guideline - Written reminder on how to access electronic guideline  Intervention theory based;  Development of intervention described in separate paper | *Professional level:* 1. Behavioural constructs 2. Behavioural simulation outcomes (vignettes) 3. X-ray & CT referral (administrative data) | Implementation strategy  led to small changes in GP intention to guideline adherence, but no statistically significant changes in actual behaviour. |
| I10. Mortimer et al. 2013 | CEA of French et al. 2013 (study I9) | See French et al. 2013 (I9) | - | *Cost of care:* Cost-effectiveness of implementation strategy versus standard dissemination from health sector perspective | Active implementation entails significant additional upfront investment that may not be offset by health gains and/or reductions in health services utilization of sufficient magnitude to render active implementation cost-effective. |
| I11. Rebbeck et al. 2006 | Cluster RCT  103 patient with whiplash 27 PTs | PT guideline: - Activation - Exercise - Multidisciplinary treatment - Pulsed electromagnetic therapy | Implementation: - Dissemination of guideline - Workshop by opinion leaders - Follow-up educational outreach visit  - Laminated copy of algorithms outlining process of care  - Appointment cards  - Marketing material  Control: No intervention  Intervention not theory based;  Development of intervention not described | *Patient level:* 1. Disability 2. Disability due to acute whiplash 3. Global Perceived Effect 4. Patient satisfaction with care from GP, care from PT, and patient version of guidelines  *Professional level:* 1. Knowledge of guidelines  2. Clinical practice 3. PT satisfaction with guidelines, intervention, and patient version of guidelines  4. Cost of care | No significant differences between groups for any of the patient outcomes at any time. Although active implementation increased guideline-consistent behaviour, cost of care was not affected. |
| I12. Schectman et al. 2003 | RCT with 4 arms  2020 patients in baseline year, 2046 patients in study year  75 physicians  21 Nurse practitioners (NPs)/Physician assistants (PAs) | GP guideline: - Imaging only in suspicion of red flags - Subspecialty referral only in suspicion of red flags - Referral to PT in lack of improvement after 6 weeks | Implementation group 1:  - 1 educational session by clinical leaders  - Copy of the guideline  - Audit report summarizing performance during baseline year, highlighting of over- and underutilization, and explaining rationale for classifications  - Individual follow-up visit (including another audit report of study year)  Implementation group 2: - All elements from implementation group 1 +  - Patient education material  - Reminder to use patient education material  Control group 1:  No intervention  Control group 2:  Patient intervention only  Intervention not theory based;  Development of intervention not described | *Professional level:* 1. Guideline consistent behaviour 2. Utilization of services  *Patient level:* 1. Beliefs about care of back pain | The 4 groups collapsed into 2 (clinician intervention vs. no clinician intervention) for analysis and reporting.  Implementation was associated with an increase in guideline consistent behaviour, but patient education materials did not enhance guideline effectiveness and were poorly adopted. |
