# Supplementary material for: Effectiveness of multifaceted implementation strategies for the implementation of back and neck pain guidelines in health care: a systematic review
Source: Implement Sci. 2016 Sep 20;11:126. doi: 10.1186/s13012-016-0482-7 (PMC5029102; doi:10.1186/s13012-016-0482-7)
Supplement: Supplementary file 4 — References to excluded full-text studies. (DOCX 17 kb) [file 13012_2016_482_MOESM4_ESM.docx]

| **Study ID** | **Clinical Practice Guidelines** | **Educational materials** | **Educational meetings** | **Educational outreach visits** | **Local opinion leaders** | **Audit & Feedback** | **Reminders** | **Other (organizational)** | **Patient mediated** | ***Total*** |
| --- | --- | --- | --- | --- | --- | --- | --- | --- | --- | --- |
| Becker et al. 2008 (I1) | X | X | X | X |  |  |  |  | X | 5 |
| Bekkering et al. 2005 (I3&I4) | X | X | X |  |  | X | X |  |  | 5 |
| Bishop et al. 2006 (I6) | X | X |  |  |  |  | X |  | X | 4 |
| Dey et al. 2004 (I7) | X | X |  | X |  |  |  | X |  | 4 |
| Engers et al. 2005 (I8) | X | X | X |  |  |  |  | X |  | 4 |
| French et al. 2013 (I9) | X | X | X |  |  |  |  |  |  | 3 |
| Rebbeck et al. 2006 (I11) | X | X | X | X | X |  |  |  |  | 5 |
| Schectman et al. 2003 I(12) | X | X | X | X | X | X |  |  | X | 7 |
| ***Total*** | 8 | 8 | 6 | 4 | 2 | 2 | 2 | 2 | 3 |  |

**Additional file 4: Table S4. Interventions of included studies according to EPOC taxonomy (all on professional level unless stated otherwise)**
