# Supplementary material for: Effectiveness of multifaceted implementation strategies for the implementation of back and neck pain guidelines in health care: a systematic review
Source: Implement Sci. 2016 Sep 20;11:126. doi: 10.1186/s13012-016-0482-7 (PMC5029102; doi:10.1186/s13012-016-0482-7)
Supplement: Supplementary file 5 — Characteristics of included studies. (DOCX 23 kb) [file 13012_2016_482_MOESM5_ESM.docx]

| **Study ID** | **Knowledge** | **Diagnostics** | **Treatment** | **Referral** | **Sickness certificates** | **Other** | **Patient outcomes** |
| --- | --- | --- | --- | --- | --- | --- | --- |
| Becker et al. 2008 (I1) |  |  |  |  |  |  | X |
| Bekkering et al. 2005 (I3&I4) |  |  | X |  |  |  | X |
| Bishop et al. 2006 (I6) |  | X | X |  |  |  |  |
| Dey et al. 2004 (I7) |  |  | X | X | X |  |  |
| Engers et al. 2005 (I8) |  |  | X | X |  |  |  |
| French et al. 2013 (I9) | X |  |  | X |  |  |  |
| Rebbeck et al. 2006 (I11) | X |  | X |  |  | Satisfaction | X |
| Schectman et al. 2003 (I12) |  |  |  | X |  |  |  |
| ***Total*** | 2 | 1 | 5 | 4 | 1 | 1 | 3 |

**Additional file 5: Table S5. Grouped outcome measures of included studies (all on professional level unless stated otherwise)**
