# Supplementary material for: Effectiveness of multifaceted implementation strategies for the implementation of back and neck pain guidelines in health care: a systematic review
Source: Implement Sci. 2016 Sep 20;11:126. doi: 10.1186/s13012-016-0482-7 (PMC5029102; doi:10.1186/s13012-016-0482-7)
Supplement: Supplementary file 6 — Appendix B. List of excluded studies and reasons for exclusion. (DOCX 17 kb) [file 13012_2016_482_MOESM6_ESM.docx]

**Additional file 6: Table S6. Summary of findings**

| **Multifaceted implementation strategies compared to usual, minimal or no implementation strategies for the implementation of neck and back pain guidelines in health care** | | | | | | |
| --- | --- | --- | --- | --- | --- | --- |
| **Patient or population**: neck and back pain guidelines  **Setting**: health care  **Intervention**: multifaceted implementation strategies  **Comparison**: usual, minimal or no implementation strategies | | | | | | |
| Outcomes | **Anticipated absolute effects^*^** (95% CI) | | Relative effect (95% CI) | № of participants  (studies) | Quality of the evidence (GRADE) | Comments |
|  | **Risk with usual, minimal or no implementation strategies** | **Risk with multifaceted implementation strategies** |  |  |  |  |
| Referral rates for X-rays follow up: range 3 months to 2 years | **Study population** | | **OR 0.93** (0.75 to 1.15) | 157175 (3 RCTs) | ⨁⨁⨁◯ MODERATE ^1^ |  |
|  | 13 per 1000 | **12 per 1000** (10 to 15) |  |  |  |  |
| Referral rates for CT/MRI scans follow up: range 12 months to 2 years | **Study population** | | **OR 0.82** (0.57 to 1.16) | 154988 (2 RCTs) | ⨁⨁◯◯ LOW ^1,2^ |  |
|  | 7 per 1000 | **6 per 1000** (4 to 9) |  |  |  |  |
| Referral rates for physiotherapy follow up: range 3 months to 2 years | **Study population** | | **OR 0.74** (0.42 to 1.32) | 4849 (3 RCTs) | ⨁◯◯◯ VERY LOW ^3,4^ |  |
|  | 505 per 1000 | **430 per 1000** (300 to 574) |  |  |  |  |
| Referral rates for secondary/specialty care follow up: range 3 months to 2 years | **Study population** | | **OR 1.16** (0.39 to 3.40) | 4233 (2 RCTs) | ⨁◯◯◯ VERY LOW ^3,4^ |  |
|  | 496 per 1000 | **533 per 1000** (277 to 770) |  |  |  |  |
| Provision of adequate patient information follow up: range 16 weeks to 12 months | **Study population** | | **OR 0.85** (0.57 to 1.27) | 1727 (3 RCTs) | ⨁⨁◯◯ LOW ^4,5^ |  |
|  | 452 per 1000 | **412 per 1000** (320 to 511) |  |  |  |  |
| Medication prescription follow up: range 16 weeks to 12 months | **Study population** | | **OR 0.87** (0.72 to 1.06) | 3414 (3 RCTs) | ⨁⨁◯◯ LOW ^3,5^ |  |
|  | 272 per 1000 | **245 per 1000** (212 to 284) |  |  |  |  |
| Advising active treatment follow up: range 16 weeks to 12 months | **Study population** | | **OR 0.69** (0.48 to 0.99) | 1727 (3 RCTs) | ⨁⨁◯◯ LOW ^3^ |  |
|  | 398 per 1000 | **313 per 1000** (241 to 396) |  |  |  |  |
| ***The risk in the intervention group** (and its 95% confidence interval) is based on the assumed risk in the comparison group and the **relative effect** of the intervention (and its 95% CI).   **CI:** Confidence interval; **OR:** Odds ratio | | | | | | |
| **GRADE Working Group grades of evidence** **High quality:** We are very confident that the true effect lies close to that of the estimate of the effect **Moderate quality:** We are moderately confident in the effect estimate: The true effect is likely to be close to the estimate of the effect, but there is a possibility that it is substantially different **Low quality:** Our confidence in the effect estimate is limited: The true effect may be substantially different from the estimate of the effect **Very low quality:** We have very little confidence in the effect estimate: The true effect is likely to be substantially different from the estimate of effect | | | | | | |
