# Supplementary material for: Effectiveness of multifaceted implementation strategies for the implementation of back and neck pain guidelines in health care: a systematic review
Source: Implement Sci. 2016 Sep 20;11:126. doi: 10.1186/s13012-016-0482-7 (PMC5029102; doi:10.1186/s13012-016-0482-7)
Supplement: Supplementary file 7 — Interventions of included studies according to EPOC taxonomy (all on professional level unless stated otherwise). (DOCX 15 kb) [file 13012_2016_482_MOESM7_ESM.docx]

**Additional file 7: Table S7. Patient outcomes at 12 months follow-up**

|  | **Outcome measure** |  |  |
| --- | --- | --- | --- |
| **Study** | **Functional capacity** | **Days of sick leave** | **Quality of life** |
|  | Mean (95% CI or SD) |  |  |
| **Becker et al. 2008**  Implementation group 1  Implementation group 2  Control | *(Hannover Functional Ability Questionnaire; 0-100%)*  72.956 (70.433-75.479)  74.637 (72.205-77.141)  71.559 (68.963-74.156) | *(Median days)*  6.159 (2.453-9.865)  6.458 (2.488-10.428)  9.271 (5.248-13.294) | *(EuroQoL)*  68.456 (66.724-70.189)  70.375 (68.649-72.100)  67.652 (65.794-69.510) |
| **Bekkering et al. 2005**  Implementation  Control | *(Quebec Back Pain Disability Scale; 0-100)*  17.0 (4.6-32.0)  13.0 (4.8-29.0) | *(Median days/previous 6 weeks)*  9.8 (30)  5.0 (15) | Not reported |
| **Rebbeck et al. 2006**  Implementation  Control | *(Functional Rating Index; 0-40)*  11.4 (8.9)  12 (10.4) | *(Days off work/past month)*  0 (0-0)  0 (0-1) | *(Core Outcome Measure (Whiplash); 1-5)*  2 (1-4)  2 (1-4) |
