# Supplementary material for: Effectiveness of multifaceted implementation strategies for the implementation of back and neck pain guidelines in health care: a systematic review
Source: Implement Sci. 2016 Sep 20;11:126. doi: 10.1186/s13012-016-0482-7 (PMC5029102; doi:10.1186/s13012-016-0482-7)
Supplement: Supplementary file 8 — Grouped outcome measures of included studies (all on professional level unless stated otherwise). (DOCX 14 kb) [file 13012_2016_482_MOESM8_ESM.docx]

**Additional file 8**

**Appendix A. Full electronic database searches**

**Medline (Pubmed):**
1. Neck pain OR back pain

"Neck Pain"[Mesh] OR "Neck Injuries"[Mesh] OR "Back Pain"[Mesh] OR ((neck[mesh] OR neck[tiab] OR cervical*[tiab] OR cervico*[tiab] OR neck[ot] OR cervical*[ot] OR cervico*[ot] OR back[mesh] OR back[tiab] OR vertebr*[tiab] OR intervertebral disk*[tiab] OR back[ot] OR vertebr*[ot] OR "Spine"[Mesh] OR spine[tiab] OR spinal[tiab] OR spine[ot] OR spinal[ot] OR intervertebral disk*[ot]) AND (pain[mesh] OR pain*[tiab] OR complaint*[tiab] OR ache*[tiab] OR disorder*[tiab] OR syndrome*[tiab] OR injury[tiab] OR injuries[tiab] OR pain*[ot] OR complaint*[ot] OR ache*[ot] OR disorder*[ot] OR syndrome*[ot] OR injury[ot] OR injuries[ot])) OR whiplash*[tiab] OR neckache*[tiab] OR mnd[tiab] OR WAD*[tiab] OR backache*[tiab] OR lbp[tiab] OR sciatica[tiab] OR lumbago[tiab] OR whiplash*[ot] OR neckache*[ot] OR mnd[ot] OR WAD*[ot] OR backache*[ot] OR sciatica[ot] OR lumbago[ot]

2. Implementation

"Health Plan Implementation"[Mesh] OR implement*[tiab] OR implement*[ot]

**Embase:**

1. Neck pain OR back pain

'neck pain'/exp OR 'neck injury'/exp OR 'backache'/exp OR whiplash*:ab,ti OR neckache*:ab,ti OR mnd:ab,ti OR WAD*:ab,ti OR backache*:ab,ti OR lbp:ab,ti OR sciatica:ab,ti OR lumbago:ab,ti OR (('neck'/exp OR neck:ab,ti OR cervical*:ab,ti OR cervico*:ab,ti OR ‘back'/exp OR back:ab,ti OR vertebr*:ab,ti OR (intervertebral NEAR/3 disk*):ab,ti OR 'spine'/exp OR spine:ab,ti OR spinal:ab,ti) AND ('pain'/exp OR pain*:ab,ti OR complaint*:ab,ti OR ache*:ab,ti OR disorder*:ab,ti OR syndrome*:ab,ti OR injury:ab,ti OR injuries:ab,ti))

2. Implementation

'health care planning'/exp OR implement*:ti,ab

**PsycINFO:**

1. Neck/back pain

DE "Back Pain" OR DE "Whiplash"

OR

TI ( whiplash* OR neckache* OR mnd OR WAD* OR backache* OR lbp OR sciatica OR lumbago ) OR AB ( whiplash* OR neckache* OR mnd OR WAD* OR backache* OR lbp OR sciatica OR lumbago )

OR

( DE "Neck (Anatomy)" OR DE "Back (Anatomy)" OR DE "Spinal Column" ) OR TI ( ( neck OR cervical* OR cervico* OR back OR vertebr* OR spine OR spinal OR “intervertebral disk” OR “intervertebral disks”) OR AB ( ( neck OR cervical* OR cervico* OR back OR vertebr* OR spine OR spinal )

AND

( DE "Pain" OR DE "Aphagia" OR DE "Back Pain" OR DE "Chronic Pain" OR DE "Neuralgia" OR DE "Neuropathic Pain" OR DE "Somatoform Pain Disorder" ) OR TI ( pain* OR complaint* OR ache* OR disorder* OR syndrome* OR injury OR injuries ) OR AB ( pain* OR complaint* OR ache* OR disorder* OR syndrome* OR injury OR injuries )

2. Implementation

TI implement* OR AB implement*

**Cochrane Library**

1. Neck pain OR back pain

whiplash* OR neckache* OR mnd OR WAD* OR backache* OR lbp OR sciatica OR lumbago OR ((neck OR cervical* OR cervico* OR back OR vertebr* OR “intervertebral disk” OR “intervertebral disks” OR Spine OR spinal) AND (pain* OR complaint* OR ache* OR disorder* OR syndrome* OR injury OR injuries))

2. Implementation

implement*

**Cinahl (Ebsco)**

1. Neck/back pain

( (MH "Back Pain+") OR (MH "Neck Pain") OR (MH "Whiplash Injuries") ) OR TI ( ( whiplash* OR neckache* OR mnd OR WAD* OR backache* OR lbp OR sciatica OR lumbago ) OR AB ( ( whiplash* OR neckache* OR mnd OR WAD* OR backache* OR lbp OR sciatica OR lumbago )

OR

( (MH "Neck") OR (MH "Back") ) OR TI ( neck OR cervical* OR cervico* OR back OR vertebr* OR spine OR spinal OR “intervertebral disk” OR “intervertebral disks ) OR AB ( neck OR cervical* OR cervico* OR back OR vertebr* OR spine OR spinal OR “intervertebral disk” OR “intervertebral disks )

AND

(MH "Pain") OR TI ( pain* OR complaint* OR ache* OR disorder* OR syndrome* OR injury OR injuries ) OR AB ( pain* OR complaint* OR ache* OR disorder* OR syndrome* OR injury OR injuries )

2. Implementation

(MH "Program Implementation") OR TI implement* OR AB implement*
