# Supplementary material for: Effectiveness of multifaceted implementation strategies for the implementation of back and neck pain guidelines in health care: a systematic review
Source: Implement Sci. 2016 Sep 20;11:126. doi: 10.1186/s13012-016-0482-7 (PMC5029102; doi:10.1186/s13012-016-0482-7)
Supplement: Supplementary file 9 — Summary of findings. (DOCX 17 kb) [file 13012_2016_482_MOESM9_ESM.docx]

**Additional file 9**

**Appendix B. List of excluded studies and reasons for exclusion**

| **Study ID** | **Excluded study** | **Reason for exclusion** |
| --- | --- | --- |
| E1 | Ammendolia et al. 2004 | Not RCT |
| E2 | Bussières et al. 2010 | Not non-specific LBP/NP |
| E3 | Bekkering et al. 2005 | Duplicate publication: Dutch summary of published results ( results included in this review) |
| E4 | Cherkin et al. 1991 | Not multifaceted |
| E5 | Cunningham et al. 2008 | Not RCT; Not guideline implementation in health care setting |
| E6 | Taramona Espinoza et al. 2012 | Not RCT; Not guideline implementation in health care setting; abstract |
| E7 | Evans et al. 2005 | Not multifaceted; study protocol |
| E8 | Fleuren et al. 2010 | Not non-specific LBP/NP; Not RCT |
| E9 | Jensen et al. 2014 | Study protocol of economic evaluation |
| E10 | Lang et al. 2002 | Not RCT |
| E11 | McKenzie et al. 2008 | Not multifaceted; study protocol |
| E12 | McKenzie et al. 2010 | Study protocol |
| E13 | Mortimer et al. 2008 | Not multifaceted; study protocol for economic evaluation of study E11 |
| E14 | Rasmussen 2002 | Not RCT; Not non-specific LBP/NP; Not guideline implementation in health care setting |
| E15 | Rebbeck et al. 2006 | Not guideline implementation in health care setting; Not RCT, Not multifaceted |
| E16 | Rebbeck et al. 2011 | Not RCT; Not guideline implementation in health care setting; abstract |
| E17 | Rebbeck et al. 2013 | Not RCT |
| E18 | Rebbeck et al. 2013 | Not RCT; Not multifaceted |
| E19 | Richings et al. 2011 | Not multifaceted, Not RCT; Not guideline implementation in health care setting; abstract |
| E20 | Riis et al. 2013 | Study protocol |
| E21 | Rossignol et al. 2000 | Not guideline implementation in health care setting |
| E22 | Rutten et al. 2011 | Not RCT; Not multifaceted; poster |
| E23 | Rutten et al. 2014 | Not RCT; Not guideline implementation in health care setting |
| E24 | Sandner-Kiesling et al. 2009 | Not multifaceted; abstract |
| E25 | Shenoy 2013 | Not peer-reviewed (dissertation) |
| E26 | Slater et al. 2014 | Not RCT |
| E27 | Stiell et al. 2009 | Not guideline implementation in health care setting |
| E28 | Suman et al. 2015 | Study protocol |
| E29 | Tracey et al. 1994 | Not RCT; Not multifaceted |
| E30 | Twomey e2003 | Not RCT; Not non-specific LBP/NP |
| E31 | Van Dulmen et al. 2014 | Not multifaceted |
