# Supplementary material for: Effectiveness of multifaceted implementation strategies for the implementation of back and neck pain guidelines in health care: a systematic review
Source: Implement Sci. 2016 Sep 20;11:126. doi: 10.1186/s13012-016-0482-7 (PMC5029102; doi:10.1186/s13012-016-0482-7)
Supplement: Supplementary file 10 — Patient outcomes at 12-month follow-up. (DOCX 14 kb) [file 13012_2016_482_MOESM10_ESM.docx]

**Additional file 10**

**Appendix C. Data sources and calculations for meta-analyses**

**Referral for X-ray:**- Dey et al. 2004; Table 3: Number of x-ray referrals in intervention and control group;
- French et al. 2013; Table 7: Number of x-ray referrals in intervention and control group;
- Schectman et al. 2003; Table 2: Number of not guideline consistent x-ray referrals in intervention year for intervention group (= group ‘clinician intervention only’ + group ‘patient/clinician intervention’: n patients in study year only) and control group (= group ‘control group’+ group ‘patient intervention only’: n patients in study year only): Intervention: 8.1*828/100=67 / Control: 8.6*1218/100=105.

**Referral for CT/MRI scan:**- French et al. 2013; Table 7: Number of CT referrals in intervention and control group;
- Schectman et al. 2003; Table 2: Number of CT or MRI referrals in intervention year for intervention group (= group ‘clinician intervention only’ + group ‘patient/clinician intervention’: n patients in study year only) and control group (= group ‘control group’+ group ‘patient intervention only’: n patients in study year only): Intervention: 3.5*828/100=29 / Control: 5.4*1218/100=66.

**Referral for PT:**- Dey et al. 2004; Table 3: Number of PT/educational programme referrals in intervention and control group. Reported as number referred, for analysis converted to number not reffered: Intervention: 1049-273=776 / Control: 1138-157=981;
- Engers et al. 2005; Table 3: Number of therapist referrals in intervention group and control group for All consultations. Reported as number referred, for analysis converted to number not reffered: Intervention: 328-75=253 / Control: 288-79=209;
- Schectman et al. 2003; Table 2: Number of PT referrals not consistent with guideline in intervention year for intervention group (= group ‘clinician intervention only’ + group ‘patient/clinician intervention’: n patients in study year only) and control group (= group ‘control group’+ group ‘patient intervention only’: n patients in study year only): Intervention: 9.2*828/100=76 / Control: 12*1218/100=146.

**Referral for specialty/secondary care:**- Dey et al. 2004; Table 3: Number of secondary care referrals in intervention and control group (Table 3). Reported as number referred, for analysis converted to number not referred: Intervention: 1049-273=776 / Control: 1138-157=981;
- Schectman et al. 2003; Table 2: Number of specialty referrals not consistent with guideline in intervention year for intervention group (= group ‘clinician intervention only’ + group ‘patient/clinician intervention’: n patients in study year only) and control group (= group ‘control group’+ group ‘patient intervention only’: n patients in study year only):
Intervention: 7.1*828/100=59 / Control: 5.6*1218/100=68.

**Provision of adequate patient information (i.e. reassuring & explaining)**
- Bekkering et al. 2005(a); Table 3: Number of patients to whom adequate information is given in intervention and control group. Reported as number adequate, for analysis converted to number inadequate: Intervention: 247-229=18 / Control: 253-221=32.
- Bishop et al. 2006(1); Figure 2A: Percentage guideline concordant education & reassurance for intervention group 2 (implementation arm 1) and control group. Reported as guideline concordant, for analysis converted to guideline disconcordant: Intervention: 90*162/100=146 / Control: 93*149/100=139.
- Bishop et al. 2006 (2); Figure 2A: Percentage guideline concordant education & reassurance for intervention group 3 (implementation arm 2) and control group. Reported as guideline concordant, for analysis converted to guideline disconcordant: Intervention: 94*151/100=142 / Control: 93*149/100=139.
- Engers et al. 2005; Table 4: Percentages of submeasures (explained no specific cause, explained pain will ease, explained no harm, explained to accept pain) of general information provided for intervention and control group for All consultations. Mean of submeasure patient numbers calculated for analysis. Reported as number of patients to whom it is provided, for analysis converted to number of patients to whom it is not provided: Intervention: 214+274+270+209=927, 927/4=242, 328-242=86 / Control: 238+246+212+180=876, 876/4=219, 288-219=69

**Medication prescription**- Bishop et al. 2006(1); Figure 2A: Percentage guideline concordant appropriate medication for intervention group 2 (implementation arm 1) and control group. Reported as guideline concordant, for analysis converted to guideline disconcordant: Intervention: 15*162/100=24 / Control: 23*149/100=34.
- Bishop et al. 2006 (2); Figure 2A: Percentage guideline concordant appropriate medication for intervention group 3 (implementation arm 2) and control group. Reported as guideline concordant, for analysis converted to guideline disconcordant: Intervention: 19*151/100=29 / Control: 23*149/100=34.
- Dey et al. 2004; Table 3: Percentage prescribed opioids or muscle relaxants for intervention and control groups.
- Engers et al. 2005; Table 3: Prescription of pain medication (general) for intervention and control groups for All consultations.

**Advising active treatment**
- Bekkering et al. 2005; Table 3: Number used mainly active interventions for intervention and control group. Reported as number adequate, for analysis converted to number inadequate: Intervention: 247-183= 64 / Control: 253-154=99.
- Bishop et al. 2006(1); Figure 2A: Percentage guideline concordant exercise for intervention group 2 (implementation arm 1) and control group. Reported as guideline concordant, for analysis converted to guideline disconcordant: Intervention: 62*162/100=100 / Control: 57*149/100=85.
- Bishop et al. 2006 (2); Figure 2A: Percentage guideline concordant exercise for intervention group 3 (implementation arm 2) and control group. Reported as guideline concordant, for analysis converted to guideline disconcordant: Intervention: 47*151/100=71 / Control: 57*149/100=85.
- Engers et al. 2005; Table 4: Percentages of submeasures (advised to stay active, advise to gradually increase activity) of activating information provided for intervention and control group for All consultations. Mean of submeasure patient numbers calculated for analysis. Reported as number of patients to whom it is provided, for analysis converted to number of patients to whom it is not provided: Intervention: 312+256=568, 568/2=284, 328-284=44 / Control: 257+188=445, 445/2=223, 288-223=65.
